# Body silhouettes as a tool to reflect obesity in the past

**DOI:** 10.1371/journal.pone.0195697

**Published:** 2018-04-25

**Authors:** Marianne Lønnebotn, Cecilie Svanes, Jannicke Igland, Karl A. Franklin, Simone Accordini, Bryndís Benediktsdóttir, Hayat Bentouhami, José A. G. Blanco, Roberto Bono, Angelo Corsico, Pascal Demoly, Shyamali Dharmage, Sandra Dorado Arenas, Judith Garcia, Joachim Heinrich, Mathias Holm, Christer Janson, Debbie Jarvis, Bénédicte Leynaert, Jesús Martinez-Moratalla, Dennis Nowak, Isabelle Pin, Chantal Raherison-Semjen, Jose Luis Sánchez-Ramos, Vivi Schlünssen, Svein Magne Skulstad, Julia Dratva, Francisco Gómez Real

**Affiliations:** 1 Department of Occupational Medicine, Haukeland University Hospital, Bergen, Norway; 2 Department of Global Public Health and Primary Care, Centre for International Health, University of Bergen, Bergen, Norway; 3 Department of Global Public Health and Primary Care, University of Bergen, Bergen, Norway; 4 Department of Surgical and Perioperative Sciences, Umeå University, Umeå, Sweden; 5 Unit of Epidemiology and Medical Statistics, Department of Diagnostics and Public Health, University of Verona, Verona, Italy; 6 Faculty of Medicine, University of Iceland, Reykjavik, Iceland; 7 Department of Respiratory Medicine and Sleep, Landspitali University Hospital, Reykjavik, Iceland; 8 Epidemiology and Social Medicine, University of Antwerp, Wilrijk, Belgium; 9 Department of Pulmonology, Universitary Hospital San Agustín, Avilés, Spain; 10 Department of Public Health and Pediatrics, University of Turin, Turin, Italy; 11 Division of Respiratory Diseases, IRCCS Policlinico San Matteo Foundation - Department of Internal Medicine and Therapeutics, University of Pavia, Pavia, Italy; 12 Department of Pulmonology, Division of Allergy, Hospital Arnaud de Villeneuve, University of Montpellier, Montpellier, France; 13 Sorbonne université, INSERM, institute Pierre-Louis d’épidémiologie et de santé publique, équipe EPAR, Paris, France; 14 Allergy and Lung Health Unit, School of Population and Global Health, The University of Melbourne, Melbourne, Australia; 15 Respiratory Department, Galdakao-Usansolo Hospital, Biscay, Spain; 16 ISGlobal, Centre for Research in Environmental Epidemiology (CREAL), Barcelona, Spain; 17 Universitat Pompeu Fabra (UPF), Barcelona, Spain; 18 CIBER Epidemiología y Salud Pública (CIBERESP), Barcelona, Spain; 19 Institute and Outpatient Clinic for Occupational, Social and Environmental Medicine, LMU München, German Center for Lung Research, München, Germany; 20 Department of Occupational and Environmental Medicine, Sahlgrenska University Hospital, Gothenburg, Sweden; 21 Department of Medical Sciences: Respiratory, Allergy & Sleep Research, Uppsala University, Uppsala, Sweden; 22 Population Health and Occupational Disease, National Heart and Lung Institute, Imperial College London, London, United Kingdom; 23 Inserm U1152, Pathophysiology and Epidemiology of Respiratory Diseases, Epidemiology Team, Paris, France; 24 University Paris Diderot Paris 7, UMR 1152, Paris, France; 25 Servicio de Neumología del Complejo, Servicio de Salud de Castilla - La Mancha, Castilla-La Mancha, Spain; 26 Facultad de Medicina de Albacete, Albacete, Universidad de Castilla - La Mancha, Castilla-La Mancha, Spain; 27 CHU Grenoble Alpes, Grenoble, France; 28 Inserm, Bordeaux Population Health Research Center, team EPICENE, UMR 1219, University Bordeaux, Bordeaux, France; 29 Department of Nursing, University of Huelva, Huelva, Spain; 30 Department of Public Health, Aarhus University, Aarhus, Denmark; 31 National Research Centre for the Working Environment, Copenhagen, Denmark; 32 Department of Immunology and Transfusion Medicine, Haukeland University Hospital, Bergen, Norway; 33 Swiss Tropical and Public Health Institute, Basel, Switzerland; 34 ZHAW, School of health professions, Winterthur, Switzerland; 35 Department of Clinical Science, University of Bergen, Bergen, Norway; 36 Department of Gynecology and Obstetrics, Haukeland University Hospital, Bergen, Norway; University College London, UNITED KINGDOM

## Abstract

Life course data on obesity may enrich the quality of epidemiologic studies analysing health consequences of obesity. However, achieving such data may require substantial resources.

We investigated the use of body silhouettes in adults as a tool to reflect obesity in the past. We used large population-based samples to analyse to what extent self-reported body silhouettes correlated with the previously measured (9–23 years) body mass index (BMI) from both measured (European Community Respiratory Health Survey, N = 3 041) and self-reported (Respiratory Health In Northern Europe study, N = 3 410) height and weight. We calculated Spearman correlation between BMI and body silhouettes and ROC-curve analyses for identifying obesity (BMI ≥30) at ages 30 and 45 years. Spearman correlations between measured BMI age 30 (±2y) or 45 (±2y) and body silhouettes in women and men were between 0.62–0.66 and correlations for self-reported BMI were between 0.58–0.70. The area under the curve for identification of obesity at age 30 using body silhouettes *vs* previously measured BMI at age 30 (±2y) was 0.92 (95% CI 0.87, 0.97) and 0.85 (95% CI 0.75, 0.95) in women and men, respectively; for previously self-reported BMI, 0.92 (95% CI 0.88, 0.95) and 0.90 (95% CI 0.85, 0.96). Our study suggests that body silhouettes are a useful epidemiological tool, enabling retrospective differentiation of obesity and non-obesity in adult women and men.

## Introduction

**Fig 1:**
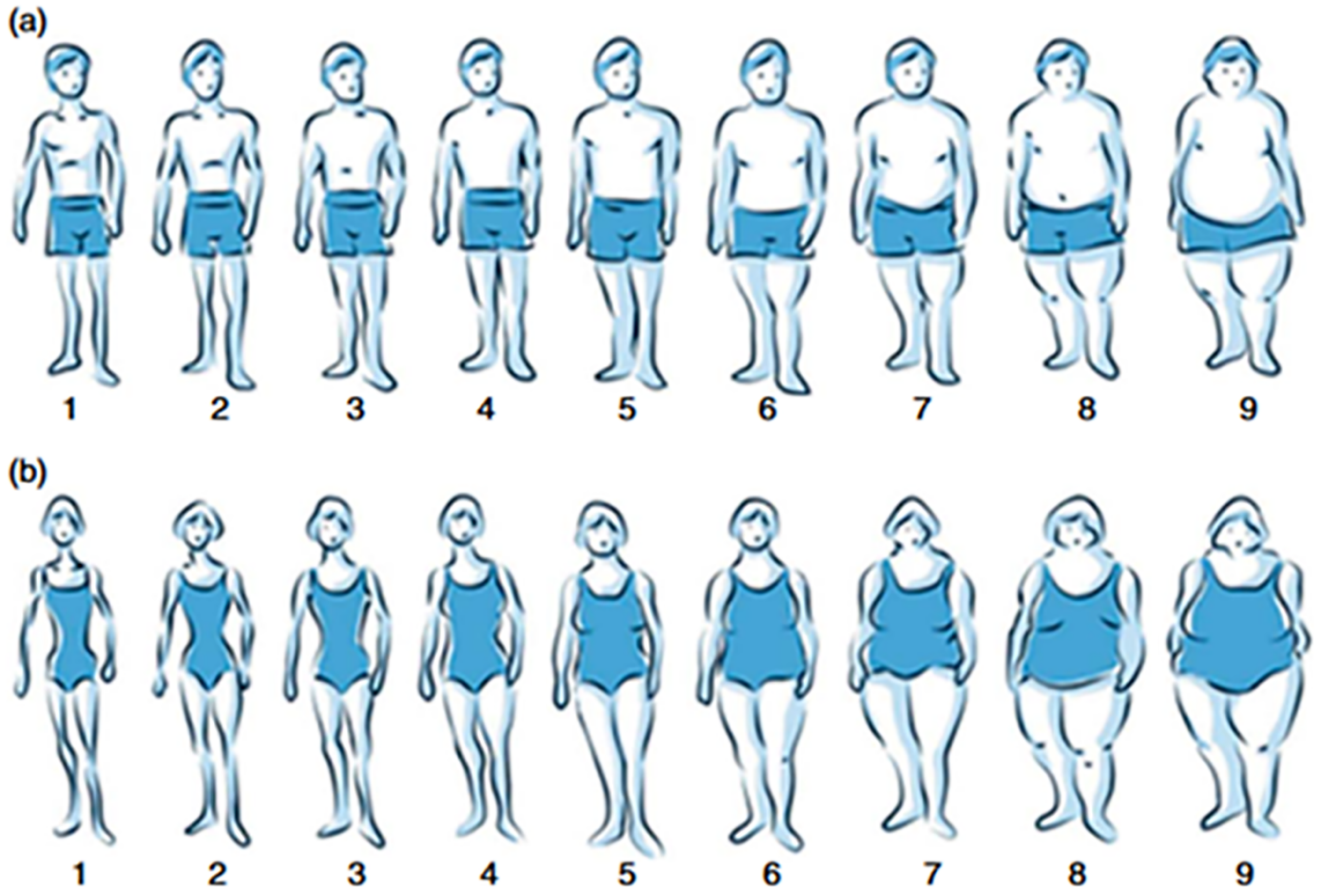
Body silhouettes for a) men and b) women introduced in the ECRHS III and RHINE III study. **a)** men, tick off your silhouette at ages: current, 8 years, voice break, 30 years, 45 years and 55 years. **b)** women, tick off your silhouette at ages: current, 8 years, menarche, 30 years, 45 years and menopause.

Obesity has emerged as one of the most prevalent risk factors for non-communicable diseases [1]. Many studies investigating the association between obesity and disease are based on simple measurements of height and weight at one time in life. Life course data on obesity may enrich the quality of epidemiologic studies of the related health consequences [2, 3]. However, many studies have not collected such data during the life course of the study participants. Figural body silhouettes from various ages might provide a possibility for retrospectively assessing obesity at several time points in the past. One possibility for assessing weight or body size in the past is to use figural body silhouettes from various ages. Sorensen et al introduced nine figural body silhouettes in 1983, from extremely lean to extremely obese, as an easy to-administer self-reported measure of body image [4]. The figural scales have been used in several studies as an adjunct to objective measured or self-reported height and weight or to assess body satisfaction [5–7]. We introduced body silhouettes (Fig 1), slightly modified from the Stunkard body images [4], in the third follow-up of the population-based cohort studies European Community Respiratory Health Survey (ECRHS) [8, 9] and Respiratory Health In Northern Europe (RHINE) study [10, 11].

Several studies have shown high correlation between objectively measured or self-reported current height and weight and body silhouettes [6, 7, 12] and we have recently reported that it is possible to define obesity and current body mass index (BMI) with high reliability from body silhouettes [13]. There is, however, little knowledge concerning the validity of assessing obesity in the past using self-reported recalled body silhouettes, beyond two small studies from selected populations assessing recall of body silhouettes, reflecting their child and adolescent years [14, 15]. Large population-based studies investigating recall of body silhouettes in adults are lacking.

We investigated the use of body silhouettes as a tool to reflect past obesity in adult men and women. We used large population-based samples to analyse to what extent self-reported body silhouettes correlated with BMI obtained from both measured and self-reported height and weight in previous surveys, conducted 9–23 years before the reporting of the body silhouettes.

## Materials and methods

### Study design

#### European Community Respiratory Health Survey

In 1991–1993, the European Community Respiratory Health Survey (ECRHS, www.ecrhs.org) first stage postal screening questionnaire was administered to population-based samples aged 20–44 years from 56 centres. The samples were randomly selected from the national population registers [8]. The ECRHS II was a follow-up study of the participants in the clinical phase of ECRHS I, performed in 29 centres between 1999 and 2002 [9]. Participants underwent the same clinical examination as in the first survey. ECRHS III is the third wave of data collection in 29 centres. It was conducted in 2011–13.

#### Respiratory Health In Northern Europe study

In seven Northern European centres (Reykjavik, Iceland; Bergen, Norway; Umeå, Uppsala and Gothenburg, Sweden; Aarhus, Denmark; and Tartu, Estonia) all responders to the first postal survey (ECRHS I) were followed at two time-points (RHINE II and RHINE III) in a large longitudinal questionnaire study, the Respiratory Health In Northern Europe study RHINE, www.rhine.nu).

### Study population

In the current study, we included persons who had A: reported their body silhouettes at age 30 or 45 in ECRHS III or RHINE III, and B: who were aged 30 (±2) years or age 45 (±2) years when they participated in previous study phases, so that they had had their height and weight measured or reported at those ages (measured in ECRHS I or II, reported in RHINE II).

We included ±2 years around age 30 and 45 to increase sample size, making the assumption that major changes in body size are unlikely to occur over two years.

**Fig 2:**
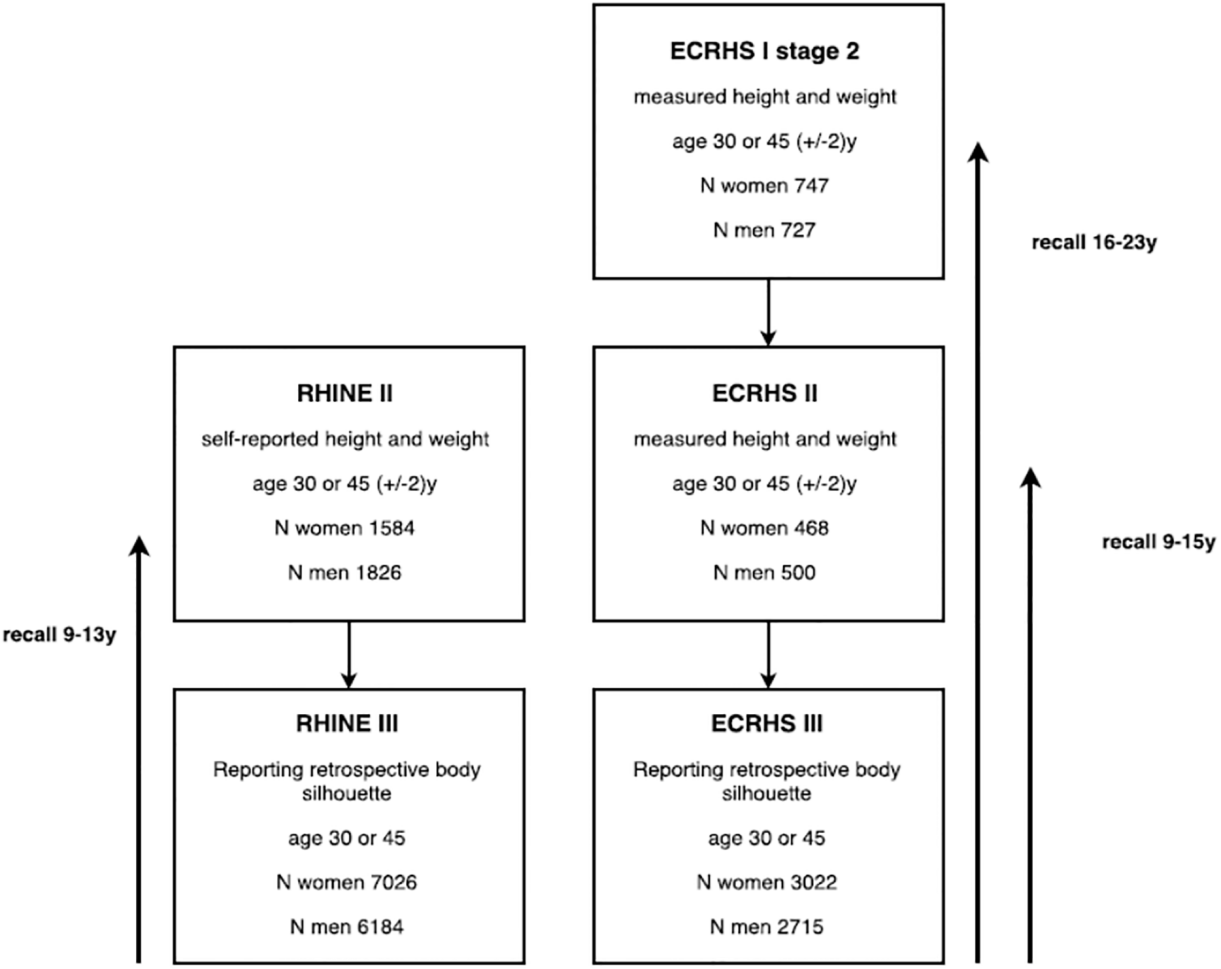
**Flow chart,** study population with participation in the RHINE III or ECRHS III study, reporting body silhouette at age 30 or 45, and with self-reported or objectively measured height and weight at age 30(±2y) or 45(±2y) in RHINE II or ECRHS I or II.

Data from ECRHS were used to validate the selected body silhouettes against BMI from measured height and weight. Among the 3 022 women and 2 715 men in ECRHS III who reported their body silhouette at age 30 or 45, 468 women and 500 men were 30 (±2) years or 45(±2) years in ECRHS II when they had their height and weight measured. In addition, 747 women and 727 men were age 30 (±2) years or 45(±2) years in ECRHS I when height and weight was measured (Fig 2). Among the 7 026 women and 6 184 men in RHINE III who reported their body silhouette at age 30 or 45, 1 584 women and 1 826 men were 30 (±2) years or 45 (±2) years in RHINE II when they reported their height and weight (Fig 2).

### Ethics

Medical research ethics committees in each study centre approved the study protocols, according to the Helsinki declaration, and all participants gave their written consent (S1 Resource).

### Body silhouettes

The figural body silhouettes (Fig 1) introduced in ECRHS III and RHINE III were designed specifically for the survey by Alejandro Villén-Real [13]. These are based on Stunkard´s body image scales [4], but slightly modified with blue colour and marked clothes. Participants were asked to tick the figural scale that best described their figure at specific time points including the ages 30 and 45 years.

### Statistical methods

All analyses were stratified by sex and validations were done separately for the body silhouettes at age 30 and 45 against objectively measured height and weight for the ECRHS participants and self-reported height and weight for the RHINE participants. The strength of the monotonic relationship between body silhouettes and BMI was estimated in terms of Spearman correlation coefficients and box plots showing median BMI and interquartile range for each body silhouette. Obesity was defined as BMI ≥ 30 kg/m^2^ according to WHO criteria. The body silhouette’s ability to classify the participants correctly according to the body mass index cut-offs was investigated by non-parametric Receiver-operating characteristic curves (ROC) and calculation of Area under the curve (AUC) with the body silhouettes specified as ordinal classification variables. The optimal cut-off was defined as the cut-off resulting from the best trade-off between specificity and sensitivity according to the Youden Index, which is defined as sensitivity + specificity -1 [16]. All analyses were performed using Stata SE 14.0.

In order to investigate how the number of recall years in the ECRHS cohort affected the association between BMI and body silhouettes, we performed additional analyses stratified on number of recall years between report of body silhouette and measurement of BMI.

## Results

**Fig 3:**
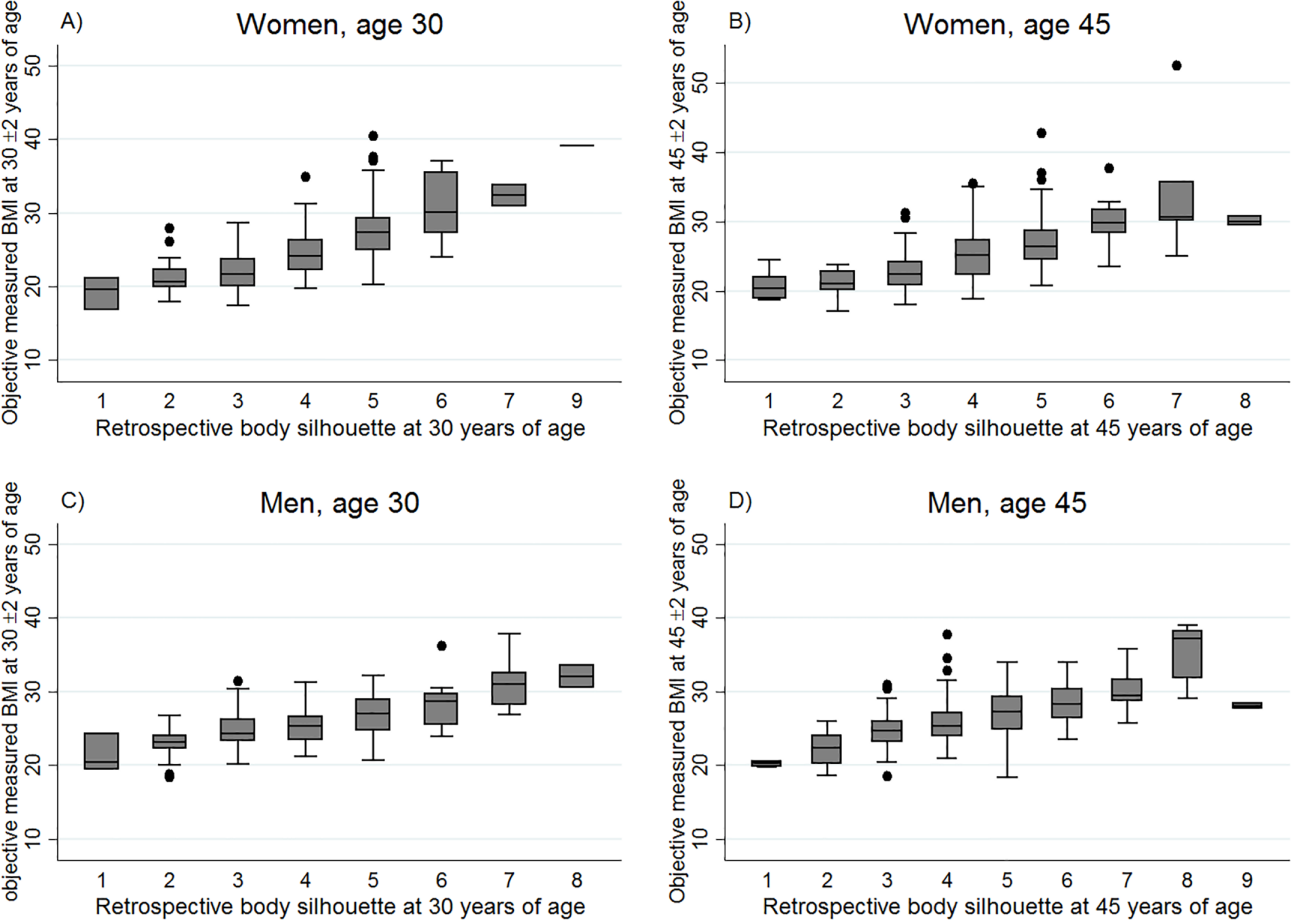
Box-and-whisker plots showing the distribution of measured BMI by figural scale (reported in ECRHS III), according to sex, in European adults aged 30(±2)years or 45(±2) years in ECRHS II,: **A)** n 172, BMI range 16.7–40.3; no reporting on figure #8; **B)** n 296, BMI range 17.0–52.5, no reporting on figure #9; **C)** n 138, BMI range 18.4–37.9, no reporting on figure #9; **D)** n 362, BMI range 18.3–39.1. The bottom and top edge of the box represent the first and third quartiles (interquartile range); the line within the box represents the median; the ends of the bottom and top whiskers represent the upper and lower adjacent values; and the dots represent outliers (ECRHS, the European Community Respiratory Health Survey).

**Fig 4:**
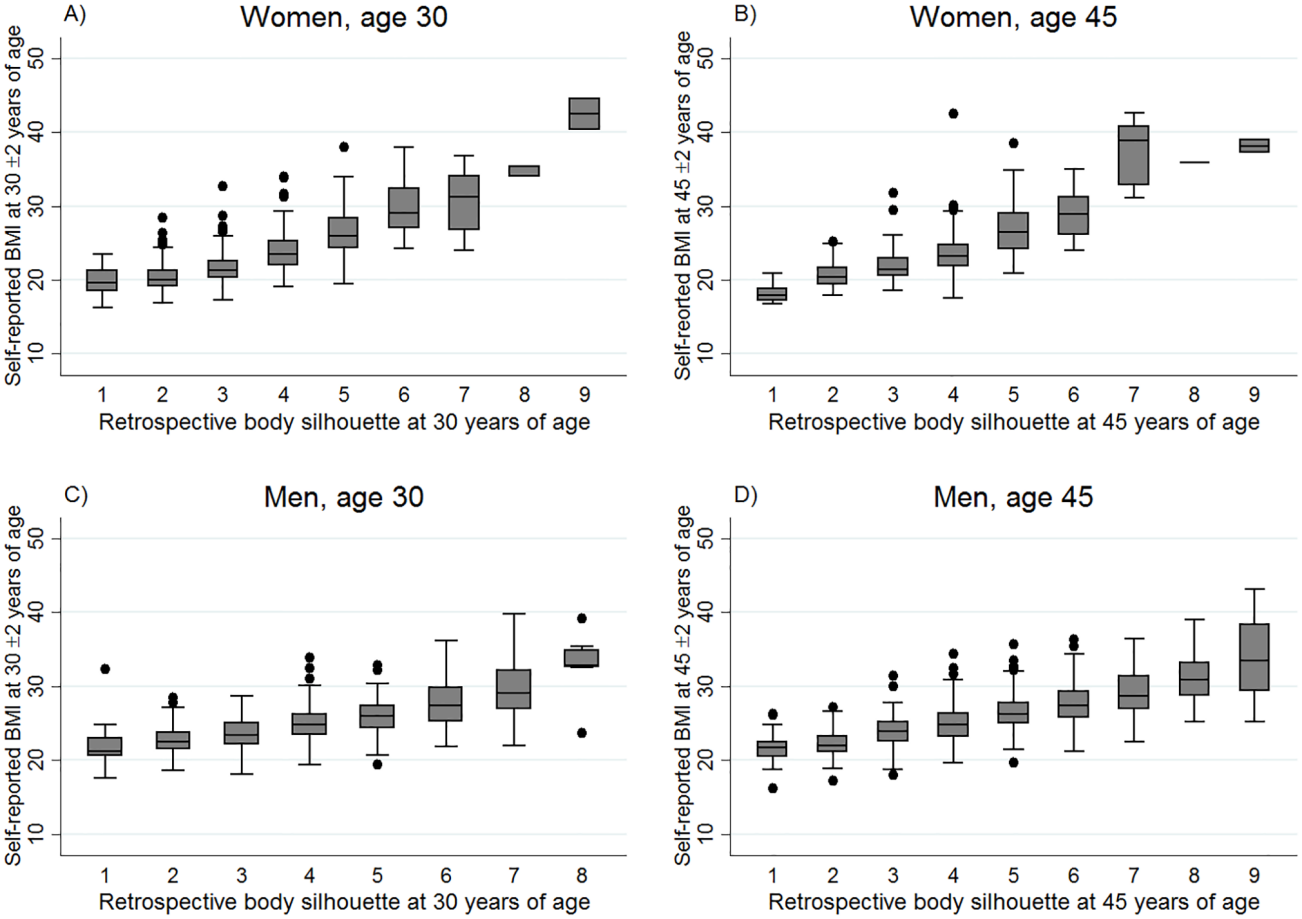
Box-and-whisker plots showing the distribution of self-reported BMI by figural scale, according to sex, in North-European adults aged 30(±2)years or 45(±2) years in RHINE II, reporting body silhouette in RHINE III: **A)** n 1145; **B)** n 439; **C)** n 829; **D)** n 997. The bottom and top edge of the box represent the first and third quartiles (interquartile range); the line within the box represents the median; the ends of the bottom and top whiskers represent the upper and lower adjacent values; and the dots represent outliers (RHINE, the Respiratory Health in Northern Europe study).

In ECRHS III 1 215 women and 1 227 men reported their recalled body silhouette at age 30 or 45 years and had their BMI measured at that age in ECRHS I or II. Mean age and BMI were slightly higher in men than in women and the median reported body silhouette in ECRHS III was silhouette #5 for both women and men (Table 1). With increasing figural scale, from scale 1 (extremely lean) to scale 9 (extremely obese), the measured BMI (median, 25^th^ and 75^th^ percentiles; measured values) increased in both women and men, and for both ages 30 and 45 years (Fig 3). The same was found for self-reported BMI (Fig 4). From body silhouette #5 and upwards, the majority of the respondents were overweight or obese according to BMI derived from height and weight measured in ECRHS I or II (S1 Fig).

**Table 1:** Characteristics of the study population reporting body silhouettes in ECRHS III and in RHINE III.

	*ECRHS III*		*RHINE III*	
	Women	Men	Women	Men
n	1215	1227	1584	1826
Age, mean (SD)	54.2 (7.5)	55.3 (7.4)	46.0 (6.6)	49.9 (7.5)
Height in meter, mean	1.63	1.77	1.68	1.81
Weight, kg, mean (SD)	71.1 (14.6)	86.3 (14.9)	69.6 (13.4)	86.7 (14.1)
BMI, mean (SD)	26.7 (5.3)	27.7 (4.4)	24.8 (4.7)	26.5 (4.0)
Current body silhouette, median	5	5	4	5

Abbreviations: ECRHS, European community respiratory health survey; RHINE, Respiratory Health In Northern Europe.

### Validation against measured BMI in women and men

Despite a recall interval of 9–15 years, there was a relatively strong correlation between self-reported past body silhouettes and previously measured BMI in women at ages 30 and 45, with a Spearman correlation of 0.64 and 0.62, respectively. With a more extended recall interval of 16–23 years, the correlation at ages 30 and 45 decreased somewhat to 0.49 and 0.57, respectively (Table 2.)

**Table 2:** Correlation between objectively measured (ECRHS I or II) or self-reported (RHINE II) height and weight and body silhouettes reported in ECRHS III and RHINE III.

*Reporting of body silhouette (BS)* *ECRHS III,* *measured BMI*	*Age*	*n*	*Mean BMI (SD)*	*Median reported BS*	Spearman correlation*	p-value**
Women						
Recall time, 9-15y	30±2y	172	24.3 (4.6)	4	0.64	p <.001
(ECRHS II)	45±2y	296	24.9 (4.4)	4	0.62	p <.001
Recall time, 16-23y	30±2y	442	22.7 (3.8)	3	0.49	p <.001
(ECRHS I)	45±2y	305	24.0 (4.1)	4	0.57	p <.001
Men						
Recall time, 9-15y	30±2y	138	25.9 (3.6)	4	0.66	p <.001
(ECRHS II)	45±2y	362	26.5 (3.7)	4	0.63	p <.001
Recall time, 16-23y	30±2y	365	24.3 (3.2)	3	0.54	p <.001
(ECRHS I)	45±2y	362	25.4 (3.3)	4	0.48	p <.001
*Reporting of body silhouette (BS)* *RHINE III*, *self-reported BMI*	*Age*	*n*	*Mean BMI (SD)*	*Median reported BS*	Spearman correlation*	p-value**
Women						
Recall time, 9-13y	30±2y	1145	23.1 (4.1)	4	0.70	p <.001
	45±2y	439	23.9 (5.0)	4	0.68	p <.001
Men						
Recall time, 9-13y	30±2y	829	24.7 (3.1)	4	0.58	p <.001
	45±2y	997	25.7 (3.3)	4	0.63	p <.001

Abbreviations: ECRHS, European community respiratory health survey; RHINE, Respiratory Health In Northern Europe; BS, Body Silhouette.

*Spearman correlations between objectively measured (ECRHS I or II) or self-reported (RHINE II) height and weight and reported body silhouettes in ECRHS III or RHINE III.

**test for significance of Spearman correlation.

The Spearman correlation between measured BMI and self-reported past body-silhouettes in men showed approximately similar results as for women, although with a higher correlation for the shorter recall time and a somewhat lower correlation for the longer recall time (Table 2).

Among obese women (BMI≥30) at age 30 years, the ROC analysis yielded an AUC value of 0.92, (95% CI 0.87, 0.97) when the recall time was 9 to 15 years (Table 3; S2 Fig), and the AUC value was 0.88, (95% CI 0.80, 0.96) when the recall time was 16–23 years (S2 Fig). In women, body silhouette #5 had the best trade-off between sensitivity and specificity for obesity at age 30 with 16% misclassification at 9–15 year recall, i.e. 16% of participants were either not obese and classified as obese or they were obese and classified as not obese. For 16–23 year recall, 12% were misclassified when body silhouette #5 was used as cut-off for classification of obesity (Table 3). The AUC for detecting obesity in women at age 45 was somewhat lower than for detecting obesity at age 30, with AUC 0.82, (95% CI 0.75, 0.89) with a recall of 9–15 years and 0.80, (95% CI 0.72, 0.89) at a recall 16–23 years. Body silhouette #5 had the best trade-off between sensitivity and specificity to detect obesity in women age 45 with a recall of 9–15 years. For a recall of 16–23 years, body silhouette #4 had the best trade-off. The misclassification was 20% at a recall of 9–15 years and 50% at a recall of 16–23 years (Table 3).

**Table 3:** Discriminatory capabilities of body silhouettes for identifying obesity (BMI≥30) retrospectively, according to sex and recall time in women and men age 30(±2) or 45(±2) years in ECRHS I or II with objectively measured height and weight and in RHINE II with self-reported height and weight. Results of ROC curve analysis.

*Reporting of body silhouette* *ECRHS III*	*Age*	*n*	*AUC* *(95% CI)*	OC*	*Youden Index*	*Sensitivity, %* *(95% CI)*	*Specificity, %* *(95% CI)*	*Correctly classified %*
*Women*								
Recall 9-15y	30±2y	172	0.92 (.87, .97)	5	.715	88.2 (.64, .99)	83.2 (.76, .89)	83.7
(ECRHS II)	45±2y	296	0.82 (.75, .89)	5	.511	72.7 (.54, .87)	78.3(.73, .83)	77.7
Recall 16-23y	30±2y	442	0.88 (.80, .96)	5	.633	75.0 (.48, .93)	88.3 (.85, .91)	87.8
(ECRHS I)	45±2y	305	0.80 (.72, .89)	4	.416	96.4 (.82, .99)	45.1 (.39, .51)	49.8
*Men*								
Recall 9–15 y	30±2y	138	0.85 (.75, .95)	6	.533	66.7 (.41, .87)	86.7(.79, .92)	84.1
(ECRHS II)	45±2y	362	0.79 (.73, .85)	5	.433	85.2 (.73, .93)	59.1(.73, .83)	63.0
Recall 16–23 y	30±2y	365	0.78 (.63, .93)	5	.548	73.3 (.45, .92)	81.4(.45, .92)	81.1
(ECRHS I)	45±2y	362	0.80 (.72, .87)	5	.463	77.4 (.82, .99)	68.9 (.64, .74)	69.6
*Reporting of body silhouette* *RHINE III*	*Age*	*n*	*AUC* *(95% CI)*	OC*	*Youden Index*	*Sensitivity*, *%* *(95% CI)*	*Specificity*, *%* *(95% CI)*	*Correctly classified %*
*Women*								
Recall 9-13y	30±2y	1145	0.92 (.88, .95)	5	.715	85.5 (.74, .93)	86.0 (.84, .88)	86.0
	45±2y	439	0.87 (.79, .94)	5	.679	85.7 (.70, .95)	82.2 (.78, .86)	82.5
*Men*								
Recall 9-13y	30±2y	829	0.90 (.85, .96)	6	.689	77.3 (.62, .89)	91.6 (.89, .93)	90.8
	45±2y	997	0.84 (.80, .88)	6	.505	67.9 (.74, .93)	82.6 (.80, .85)	81.4

Abbreviations: AUC, Area Under the Curve; BMI, Body Mass Index; ECRHS, European community respiratory health survey; RHINE, Respiratory Health In Northern Europe.

*OC, optimal sensitivity and specificity criterion in relation to body silhouette.

In men, the AUC values for detecting obesity at age 30 were 0.85, (95% CI 0.75, 0.95) with a recall time of 9–15 years, and 0.78, (95% CI 0.63, 0.93) for a recall time of 16–23 years (Table 3; S2 Fig). For 9–15 year recall, body silhouette #6 had the best trade-off between sensitivity and specificity for obesity, with a 16% misclassification. For 16–23 year recall, body silhouette #5 had the best cut-off, with 19% misclassification (Table 3).

To detect obesity in men at age 45, the AUC did not vary by recall time, AUC 0.79, (95% CI 0.73, 0.85) with a recall of 9–15 years and AUC 0.80, (95% CI 0.72, 0.87) with at recall time of 16–23 years (Table 3). Body silhouette #5 had the best trade-off between sensitivity and specificity to detect obesity in men age 45, regardless of recall time, with a misclassification of 37–40% (Table 3).

### Validation against self-reported BMI in women and men

Validating past body silhouettes against self-reported height/weight data gave approximately similar results as the validation against measured height/weight. In RHINE III 1 584 women and 1 826 men reported their historical body silhouette at age 30 or 45 years and had 9–13 years earlier self-reported their height and weight in RHINE II. In RHINE III mean age and BMI was higher in men than in women and median reported body silhouette was #4 for women and #5 for men (Table 1). For women, the Spearman correlation between self-reported BMI in RHINE II and reporting of body silhouette age 30 (RHINE III), was 0.70. At age 45, spearman correlation was 0.68 (Table 2). For men, the spearman correlation was 0.58 between self-reported BMI (RHINE II), and reporting of body silhouette age 30 (RHINE III), and 0.68 for age 45. The recall time was 9–13 years in the RHINE study (Table 2).

In women, the AUC from ROC curve analyses was 0.92, (95% CI 0.88, 0.95) at age 30 (±2y), and 0.87, (95% CI 0.79–0.94) at age 45(±2y). Body silhouette #5 had the best trade-off between sensitivity and specificity for obesity at ages 30 and 45 years, with 14% and 17% respectively, being misclassified (Table 3).

For men, the AUC from ROC curve analyses were 0.90, (95% CI 0.85, 0.96) at age 30(±2y), and 0.84, (95% CI 0.80, 0.88) at age 45(±2y). Body silhouette #6 had the best trade-off between sensitivity and specificity for obesity at ages 30 and 45 years, with 9% and 18%, respectively, being misclassified (Table 3).

## Discussion

This analysis of large population-based cohorts of adult women and men showed that reported past body silhouettes correlated with BMI as measured (reported) at the corresponding ages, and that the retrospective body silhouettes made it possible to differentiate between obese and non-obese persons at previous ages with an acceptable validity. Longer recall times weakened the correlations to some extent, while the age at the time of recalled body size was of larger importance for detecting obesity. These results were the same for both women and men, and the findings were consistent when validated against measured as well as self-reported anthropometric data.

This is the first study to validate the use of self-reported body silhouettes against previously measured or self-reported height and weight during adulthood in a large study population, and to study the importance of recall time. The concept that body silhouettes may be a valid tool to recall body size back in time is supported by two previous studies. Must et al [14] asked elderly men and women aged 71–76 years (n 181) to select body silhouettes reflecting their childhood and adolescent years. With a recall time of several decades they found that selected body sizes were reasonably well correlated with measured BMI age 20 (r^Pearsson^ = 0.51 in men and 0.64 in women). A study by Koprowski et al [15] investigated the ability of women with an average age of 17 years (n = 132) to recall body size at the age of menarche using body silhouettes, and found good correlation between actual BMI at the time of menarche and body silhouette (r^Pearsson^ = 0.77). Thus, good correlation between body silhouettes and measured height and weight is a consistent finding in these studies of elderly and adolescent body size and our study of adult body sizes.

In our study there were no significant gender differences, but the discriminatory capability of the body silhouettes to identify obesity was slightly better in women than in men, both with regard to measured and self-reported BMI. Must et al [14] also suggested a somewhat stronger correlation in women, and they found gender differences in over- *vs* underestimation of high school weight. There is no previous literature addressing potential differences relating to recall time. In our analysis, the associations of recalled body silhouette with measured/reported height and weight did not significantly differ according to recall time, although correlations appeared somewhat weaker for the longer recall time. This agrees with the findings of Must et al [14] of overall moderate correlations even in the remote past. However, our analyses suggest that obesity was better discriminated at age 30 years than at age 45 years, a higher percentage of both women and men were “correctly classified” as obese at age 30. We speculate that this could be related to age 45 years being an age of transition and a more unstable weight.

The body silhouettes showed the best discriminatory capability for obese women and men age 30(±2y) with short recall time. The ROC curve analyses showed that for both women and men age 30(±2y), more than 80% were correctly classified when the optimal cut-off value according to the Youden index was applied. For some groups, however, the sensitivities were quite low when the optimum cut-off was used. There is therefore, no support for a common cut-off that could be used to accurately separate between obese and non-obese participants, for both genders and all ages. There was an overlap of the BMI- range associated with each body silhouette, as the body silhouettes represent gradations in body size. In general, the retrospective body silhouettes are best used as an ordinal measure of body size and probability of obesity, rather than a measure to strictly define BMI groups.

The differences in associations with measured and self-reported height and weight were minimal. We observed a slightly higher percentage of correctly classified individuals using self-reported data than when using objectively measured height and weight. This might be due to the fact that both body silhouettes and weight and height were self-reported in RHINE, and participants who have a tendency to over- or under-report past body size might possibly also have a tendency to previously have over- or under-reported weight.

This study’s strong point is the high numbers of participant with data on measured or self-reported height and weight at two time points in the past, in both women and men. Participants were originally recruited randomly from the general population living at the respective study sites. The multi-centre design is also a strong asset. Our results should thereby be generalizable to adult women and men living in areas comparable to the investigated study sites in Europe and Melbourne, Australia. The tool would need to be validated for use in other populations.

As to the limitations of the self-reported body silhouettes, individual differences in misconception of body size cannot be captured by our analysis. Further, there has been some criticism of the coarse and ordinal nature of the body silhouette scale. Their ordinal and fixed scale forces people to decide on one figure or the other, even though they might feel they are in between two figural scales. This may contribute to the variation in BMI within each figural scale [17, 18].

Most studies investigating the association between obesity and disease are based on single measurements of height and weight at one time in life. Life course data on obesity may enrich the quality of epidemiologic studies of the related health consequences [2, 3]. In the absence of previously measured height and weight, alternative methods for assessment are needed that can capture key features of weight history. The use of body silhouettes through the lifespan may be a simple and inexpensive epidemiological tool to obtain this information.

In conclusion, our study suggests that body silhouettes can be a satisfactory correlate for past body size in adulthood and a useful epidemiological tool to differentiate retrospectively between non-obesity or obesity in women and men. There was no specific cut-off for the body silhouettes that can be used to define obese persons, but the probability of being obese increased with increasing body silhouette.

## Supporting information

S1 Fig **Distribution of retrospectively self-reported body silhouettes in relation to measured height and weight in ECRHS I or II** (different **colours** illustrating the different BMI-categories; y-axis showing percent of BMI-category in each bar; x-axis showing the different body silhouettes by number).
(TIF)

S2 Fig **ROC-curves, discriminatory capabilities of body silhouettes for identifying obesity retrospectively, according to sex and recall time in women and men age 30(±2) years in the European Community Respiratory Health Survey (ECRHS I/II) with objectively measured height and weight; A)** women 30(±2)y, recall time 9–15 y, sensitivity = 0.88, specificity = 0.83, AUC = 0.92; **B)** women 30(±2)y, 16-23y recall time, sensitivity = 0.75, specificity = 0.88, AUC = 0.88; **C)** men 30(±2)y, recall time 9-15y, sensitivity = 0.67, specificity = 0.87, AUC = 0.85; **D)** men 30(±2)y, 16-23y recall time, sensitivity = 0.73, specificity = 0.81, AUC = 0.78 (ROC, receiver-operating characteristic; AUC, area under the curve).
(TIF)

S1 Resource Ethics committees and approval numbers ECRHS/RHINE. (DOCX)

S2 Resource Funding. Sources for the local ECRHS and RHINE studies.
(DOCX)
