# Supplementary figures and images for: Body silhouettes as a tool to reflect obesity in the past
Source: PLoS One. 2018 Apr 25;13(4):e0195697. doi: 10.1371/journal.pone.0195697 (PMC5918897; doi:10.1371/journal.pone.0195697)

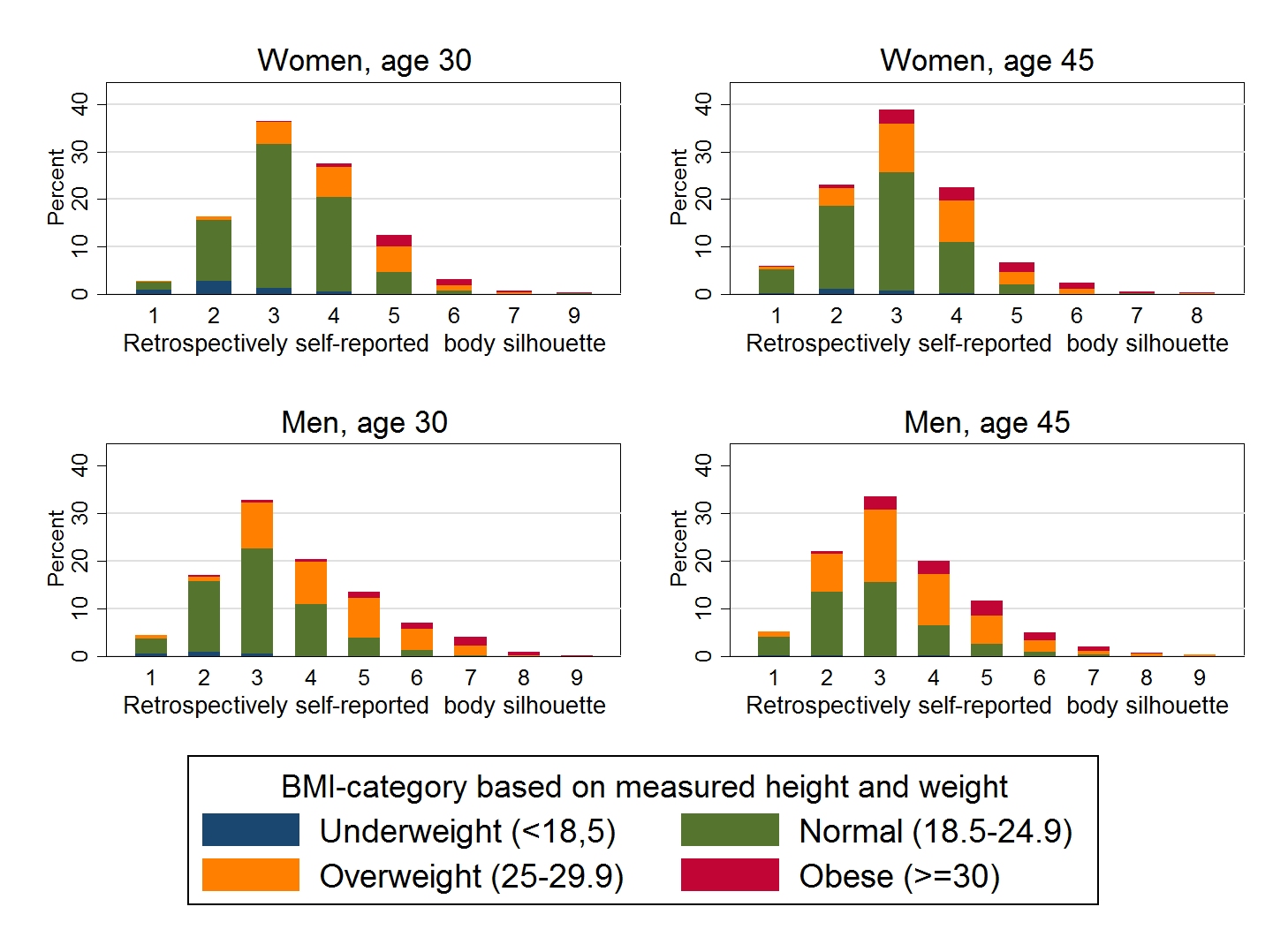

Supplement: S1 Fig — Distribution of retrospectively self-reported body silhouettes in relation to measured height and weight in ECRHS I or II (different colours illustrating the different BMI-categories; y-axis showing percent of BMI-category in each bar; x-axis showing the different body silhouettes by number). (TIF) [file pone.0195697.s001.tif]

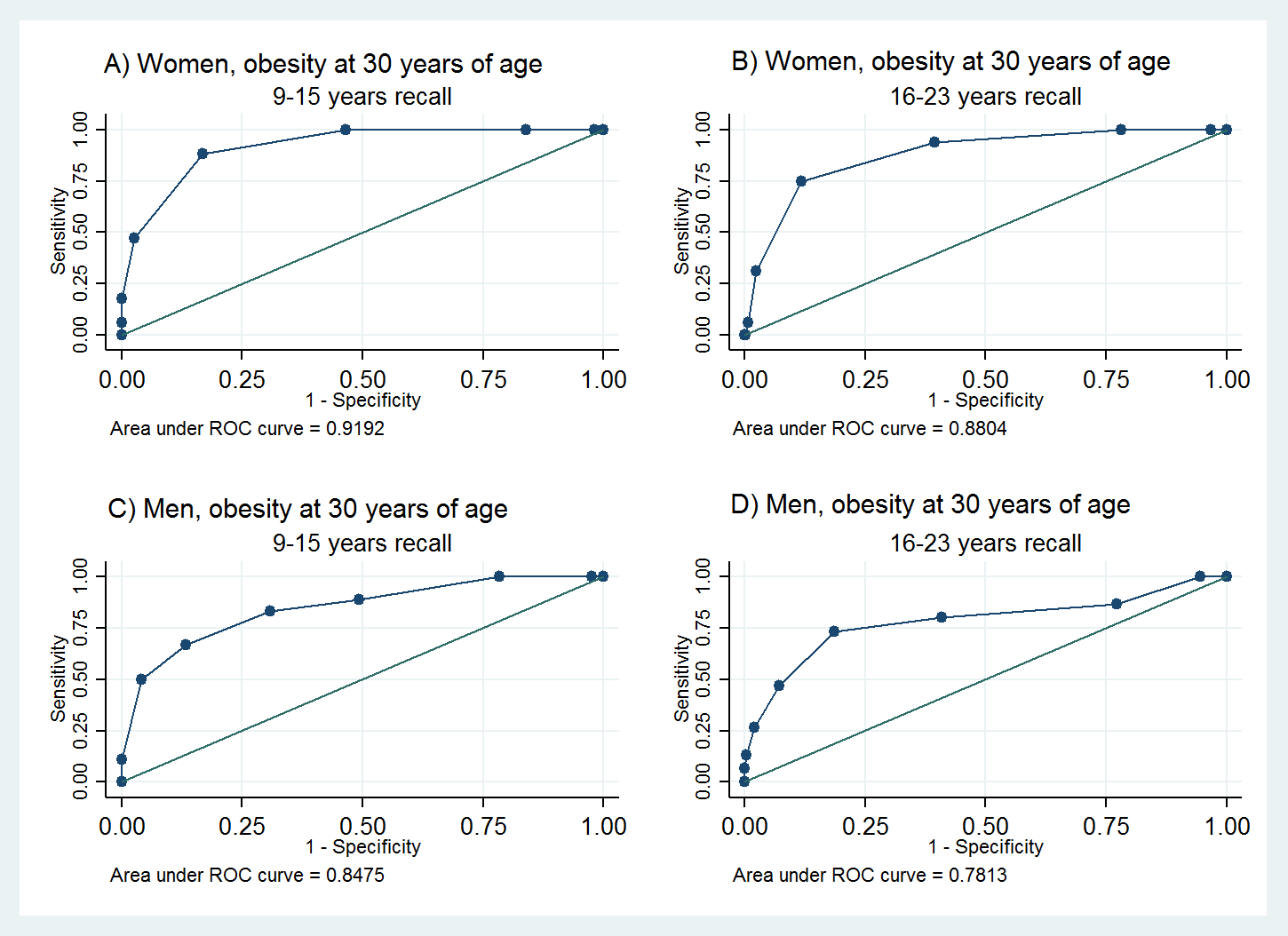

Supplement: S2 Fig — ROC-curves, discriminatory capabilities of body silhouettes for identifying obesity retrospectively, according to sex and recall time in women and men age 30(±2) years in the European Community Respiratory Health Survey (ECRHS I/II) with objectively measured height and weight; A) women 30(±2)y, recall time 9–15 y, sensitivity = 0.88, specificity = 0.83, AUC = 0.92; B) women 30(±2)y, 16-23y recall time, sensitivity = 0.75, specificity = 0.88, AUC = 0.88; C) men 30(±2)y, recall time 9-15y, sensitivity = 0.67, specificity = 0.87, AUC = 0.85; D) men 30(±2)y, 16-23y recall time, sensitivity = 0.73, specificity = 0.81, AUC = 0.78 (ROC, receiver-operating characteristic; AUC, area under the curve). (TIF) [file pone.0195697.s002.tif]
