# Supplementary material for: Body silhouettes as a tool to reflect obesity in the past
Source: PLoS One. 2018 Apr 25;13(4):e0195697. doi: 10.1371/journal.pone.0195697 (PMC5918897; doi:10.1371/journal.pone.0195697)
Supplement: S1 Resource — (DOCX) [file pone.0195697.s003.docx]

Ethical approvals ECRHS/RHINE

| **Study centres** | **Committee name and approval number** |
| --- | --- |
| Australia, Melbourne | Monash University Human Research Ethics Committee Project # CF11/1818-2010001012 |
| Belgium, South Antwerp and Antwerp City | Comité voor Medische Ethiek UZA/UA 11/41/288 – UA |
| Denmark, Aarhus | De Videnskabsetiske Komiteer for region Midtjylland.  M-20110106 |
| Estonia, Tartu | Research Ethics Committee of the University of Tartu (UT REC) 209T-17 and 225/M-24 |
| France, Paris | Etude ECRHS III: promotion CHU de Grenoble. Ethical approval CPP Sud est V 4 mars 2011. Approval Ministry of Health AFSSAPS n°B110053-70. |
| France, Grenoble | Etude ECRHS III: promotion CHU de Grenoble. Ethical approval CPP Sud est V 4 mars 2011. Approval Ministry of Health AFSSAPS n°B110053-70. |
| France, Montpellier | Etude ECRHS III : promotion CHU de Grenoble. Ethical approval CPP Sud est V 4 mars 2011. Approval Ministry of Health AFSSAPS n°B110053-70. |
| France, Bordeaux | Etude ECRHS III : promotion CHU de Grenoble. Ethical approval CPP Sud est V 4 mars 2011. Approval Ministry of Health AFSSAPS n°B110053-70. |
| Germany, Hamburg | Ethikkommission der Bayerischen Landesärztekammer (Positive Votum: 10015 |
| Germany, Erfurt | Ethikkommission der Bayerischen Landesärztekammer (Positive Votum: 10015 |
| Iceland, Reykjavik | National Bioethics committee of Iceland VSN-11-121-S3 |
| Norway, Bergen | Regional Ethics Committee West Norway 2010/759 |
| Spain, Barcelona | Ethics Committee of the Parc de Salut Mar, Barcelona (Comité del etico d’investigacion clínica (CEIC)- Parc de Salut Mar, Barcelona ( Approval number) 2009/3500/1 |
| Spain, Galdakao | Ethics Committee of the Parc de Salut Mar, Barcelona (Comité etic d’investigacio clínica (CEIC)- Parc de Salut Mar, Barcelona ( Approval num) 2009/3500/1 |
| Spain, Albacete | Ethics Committee of the Parc de Salut Mar, Barcelona (Comité etic d’investigacio clínica (CEIC)- Parc de Salut Mar, Barcelona ( Approval number) 2009/3500/1 |
| Spain, Oviedo | Ethics Committee of the Parc de Salut Mar, Barcelona (Comité del etico d’investigacion clínica (CEIC)- Parc de Salut Mar, Barcelona ( Approval number) 2009/3500/1 |
| Spain, Huelva | Ethics Committee of the Parc de Salut Mar, Barcelona (Comité del etico d’investigacion clínica (CEIC)- Parc de Salut Mar, Barcelona ( Approval number) 2009/3500/1 |
| Sweden, Gothenburg | Regional Ethical Review Board in Uppsala. 2010/432 |
| Sweden, Uppsala | Regional Ethical Review Board in Uppsala. And the number of the decision is 2010/432 |
| Sweden, Umeaa | Regional Ethical Review Board in Uppsala. 2010/432 |
| UK, Ipswich | NRES committee London-Stanmore REC Ref: 11/LO/0965 |
| UK, Norwich | NRES committee London-Stanmore REC Ref: 11/LO/0965 |
