# Supplementary material for: Body silhouettes as a tool to reflect obesity in the past
Source: PLoS One. 2018 Apr 25;13(4):e0195697. doi: 10.1371/journal.pone.0195697 (PMC5918897; doi:10.1371/journal.pone.0195697)
Supplement: S2 Resource — Sources for the local ECRHS and RHINE studies. (DOCX) [file pone.0195697.s004.docx]

**FUNDING**

**Financial support for RHINE**:

**Denmark**: The Faculty of Health of Aarhus University (Project No. 240008), The Wood Dust Foundation (Project No. 444508795), The Danish Lung Association. **Estonia**: The Estonian Science Foundation (Grant No. 4350). **Iceland**: The Icelandic Research Council. **Norway**: Research Council of Norway (Grants No. 214123, 230827and 228174), the Bergen Medical Research Foundation, the Western Norwegian Regional Health Authorities (Grants No. 912011, 911892 and 911631), the Norwegian Labour Inspection and the Norwegian Asthma and Allergy, **Sweden**: The Swedish Heart and Lung Foundation, the Vårdal Foundation for Health Care Science and Allergy Research, the Swedish Council for Working Life and Social Research, the Bror Hjerpstedt Foundation, the Swedish Asthma and Allergy Association.

**Financial support for ECRHS I:**

The co-ordination of ECRHS I was supported by the European Commission

The following grants helped to fund the local studies.

**Australia:** Asthma Foundation of Victoria, Allen and Hanbury's, **Belgium**: Belgian Science Policy Office, National Fund for Scientific Research, **Denmark: Aarhus** ( R.Dahl, M.Iversen) **Estonia:** Estonian Science Foundation, grant no 1088, **France:** Ministère de la Santé, Glaxo France, Insitut Pneumologique d'Aquitaine, Contrat de Plan Etat-Région Languedoc-Rousillon, CNMATS, CNMRT (90MR/10, 91AF/6), Ministre delegué de la santé, RNSP, France; GSF, **Germany**: Bundesminister für Forschung und Technologie, **Greece:** The Greek Secretary General of Research and Technology, Fisons, Astra and Boehringer-Ingelheim; **Italy:** Ministero dell'Università e della Ricerca Scientifica e Tecnologica, CNR, Regione Veneto grant RSF n. 381/05.93, **Netherlands** Dutch Ministry of Wellbeing, Public Health and Culture and the Netherlands Asthma Foundation, **Norway:** Norwegian Research Council project no. 101422/310; **Portugal:** Glaxo Farmacêutica Lda, Sandoz Portugesa, **Spain:** Fondo de Investigación Sanitaria ( #91/0016-060-05/E, 92/0319 and #93/0393), Hospital General de Albacete, Hospital General Juan Ramón Jiménez, Dirección Regional de Salud Pública (Consejería de Sanidad del Principado de Asturias), CIRIT (1997 SGR 00079) and Servicio Andaluz de Salud; **Sweden:** The Swedish Medical Research Council, the Swedish Heart Lung Foundation, the Swedish Association against Asthma and Allergy; **Switzerland:** Swiss national Science Foundation grant 4026-28099; **UK:** National Asthma Campaign, British Lung Foundation, Department of Health, South Thames Regional Health Authority**,**

**ECRHS II**

The co-ordination of ECRHS II was supported by the European Commission

The following grants helped to fund the local studies.

**Australia:** National Health and Medical Research Council**, Belgium:** Antwerp: Fund for Scientific Research (grant code, G.0402.00), University of Antwerp, Flemish Health Ministry**;** **Estonia: Tartu** Estonian Science Foundation grant no 4350, **France: (All)** Programme Hospitalier de Recherche Clinique—Direction de la Recherche Clinique (DRC) de Grenoble 2000 number 2610, Ministry of Health, Ministère de l’Emploi et de la Solidarité, Direction Génerale de la Santé, Centre Hospitalier Universitaire (CHU) de Grenoble, **Bordeaux**: Institut Pneumologique d’Aquitaine; **Grenoble:** Comite des Maladies Respiratoires de l’Isere **Montpellier:** Aventis ( France), Direction Regionale des Affaires Sanitaires et Sociales Languedoc-Roussillon**; Paris:** Union Chimique Belge- Pharma (France),Aventis (France), Glaxo France, **Germany: Erfurt** GSF—National Research Centre for Environment and Health, Deutsche Forschungsgemeinschaft (grant code, FR1526/1-1) **Hamburg:** GSF—National Research Centre for Environment and Health, Deutsche Forschungsgemeinschaft (grant code, MA 711/4-1) **Iceland: Reykjavik**, Icelandic Research Council, Icelandic University Hospital Fund; **Italy: Pavia** GlaxoSmithKline Italy, Italian Ministry of University and Scientific and Technological Research (MURST),Local University Funding for Research 1998 and 1999;**Turin**: Azienda Sanitaria Locale 4 Regione Piemonte (Italy), Azienda Ospedaliera Centro Traumatologico Ospedaliero/Centro Traumatologico Ortopedico—Istituto Clinico Ortopedico Regina Maria Adelaide Regione Piemonte **Verona:** Ministero dell’Universita´ e della Ricerca Scientifica (MURST), Glaxo Wellcome spa, **Norway: Bergen:** Norwegian Research Council, Norwegian Asthma and Allergy Association, Glaxo Wellcome AS, Norway Research Fund; **Spain:** Fondo de Investigacion Santarias (grant codes, 97/0035-01,99/0034-01 and 99/0034 02), HospitalUniversitario de Albacete, Consejeria de Sanidad; **Barcelona:** Sociedad Espanola de Neumologı´a y Cirugı´a Toracica, Public Health Service(grant code, R01 HL62633-01), Fondo de Investigaciones Santarias (grant codes, 97/0035-01, 99/0034-01, and 99/0034-02), Consell Interdepartamentalde Recerca i Innovacio´ Tecnolo`gica (grant code, 1999SGR 00241) Instituto de Salud Carlos III; Red deCentros de Epidemiologı´a y Salud Pu´blica, C03/09,Redde Basesmoleculares y fisiolo´gicas de lasEnfermedadesRespiratorias,C03/011and Red de Grupos Infancia y Medio Ambiente G03/176; **Huelva:** Fondo de Investigaciones Santarias (grant codes, 97/0035-01, 99/0034-01, and 99/0034-02); **Galdakao:** Basque Health Department **Oviedo:** Fondo de Investigaciones Sanitaria (97/0035-02, 97/0035, 99/0034-01, 99/0034-02, 99/0034-04, 99/0034-06, 99/350, 99/0034--07), European Commission (EU-PEAL PL01237), Generalitat de Catalunya (CIRIT 1999 SGR 00214), Hospital Universitario de Albacete, Sociedad Española de Neumología y Cirugía Torácica (SEPAR R01 HL62633-01) Red de Centros de Epidemiología y Salud Pública (C03/09), Red de Bases moleculares y fisiológicas de las Enfermedades Respiratorias (C03/011) and Red de Grupos Infancia y Medio Ambiente (G03/176);97/0035-01, 99/0034-01, and99/0034-02); **Sweden: Göteborg , Umea, Uppsala**: Swedish Heart Lung Foundation, Swedish Foundation for Health Care Sciences and Allergy Research, Swedish Asthma and Allergy Foundation, Swedish Cancer and Allergy Foundation, Swedish Council for Working Life and Social Research (FAS),**Switzerland: Basel** Swiss National Science Foundation, Swiss Federal Office for Education and Science, Swiss National Accident Insurance Fund; **UK: Ipswich and Norwich**: Asthma UK (formerly known as National Asthma Campaign)

**ECRHS III**

The co-ordination of ECRHS III was supported by the Medical Research Council (Grant Number 92091)

The following grants helped to fund the local studies.

**Australia**: National Health & Medical Research Council**, Belgium: Antwerp South, Antwerp City:** Research Foundation Flanders (FWO), grant code G.0.410.08.N.10 (both sites), **Estonia: Tartu-** SF0180060s09 from the Estonian Ministry of Education. **France:** (**All**) Ministère de la Santé. Programme Hospitalier de Recherche Clinique (PHRC) national 2010. **Bordeaux:** INSERM U897 Université Bordeaux segalen, **Grenoble:** Comite Scientifique AGIRadom 2011. **Paris:** Agence Nationale de la Santé, Région Ile de France, domaine d’intérêt majeur (DIM) **Germany : Erfurt:** German Research Foundation HE 3294/10-1 **Hamburg**: German Research Foundation MA 711/6-1, NO 262/7-1 **Iceland:** Reykjavik, The Landspitali University Hospital Research Fund, University of Iceland Research Fund, ResMed Foundation, California, USA, Orkuveita Reykjavikur (Geothermal plant), Vegagerðin (The Icelandic Road Administration (ICERA). **Italy:** All Italian centres were funded by the Italian Ministry of Health, Chiesi Farmaceutici SpA, in addition **Verona** was funded by Cariverona foundation, Education Ministry (MIUR). **Norway:** Norwegian Research council grant no 214123, Western Norway Regional Health Authorities grant no 911631, Bergen Medical Research Foundation.  **Spain:** Fondo de Investigación Sanitaria (PS09/02457, PS09/00716 09/01511) PS09/02185 PS09/03190), Servicio Andaluz de Salud , Sociedad Española de Neumología y Cirurgía Torácica (SEPAR 1001/2010); **Sweden:** All centres were funded by The Swedish Heart and Lung Foundation, The Swedish Asthma and Allergy Association, The Swedish Association against Lung and Heart Disease.Fondo de Investigación Sanitaria (PS09/02457 **Barcelona:**Fondo de Investigación Sanitaria (FIS PS09/00716) Galdakao: Fondo de Investigación Sanitaria (FIS 09/01511) **Huelva**: Fondo de Investigación Sanitaria (FIS PS09/02185) and Servicio Andaluz de Salud **Oviedo:** Fondo de Investigación Sanitaria (FIS PS09/03190) **Sweden:** All centres were funded by The Swedish Heart and Lung Foundation, The Swedish Asthma and Allergy Association, The Swedish Association against Lung and Heart Disease. **Swedish Research Council for health, working life and welfare (FORTE) Göteborg** **:** Also received further funding from the Swedish Council for Working life and Social Research. Umea also received funding from Vasterbotten Country Council ALF grant. **Switzerland:** The Swiss National Science Foundation (grants no 33CSCO-134276/1, 33CSCO-108796, 3247BO-104283, 3247BO-104288, 3247BO-104284, 3247-065896, 3100-059302, 3200-052720, 3200-042532, 4026-028099) The Federal office for forest, environment and landscape, The Federal Office of Public Health, The Federal Office of Roads and Transport, the canton’s government of Aargan, Basel-Stadt, Basel-Land, Geneva, Luzern, Ticino, Valais and Zürich, the Swiss Lung League, the canton's Lung League of Basel Stadt/ Basel, Landschaft, Geneva, Ticino, Valais and Zurich, SUVA, Freiwillige Akademische Gesellschaft, UBS Wealth Foundation, Talecris Biotherapeutics GmbH, Abbott Diagnostics, European Commission 018996 (GABRIEL), Wellcome Trust WT 084703MA, **UK:** Medical Research Council (Grant Number 92091). Support also provided by the National Institute for Health Research through the Primary Care Research Network
